# Medication Administration in Poststroke Dysphagia: Evaluating Swallowing Safety of Solid Dosage Forms

**DOI:** 10.1161/STROKEAHA.125.051237

**Published:** 2025-07-16

**Authors:** Michaela Trapl-Grundschober, Walter Struhal, Yvonne Teuschl, Steffen Schulz, Simon Sollereder, Jürgen Osterbrink

**Affiliations:** Karl Landsteiner University of Health Sciences, Krems, Lower Austria, Austria (M.T.-G., W.S.).; Division of Neurology, University Hospital Tulln, Lower Austria, Austria (M.T.-G., W.S.).; Doctor of Philosophy (Ph.D.) Program, Institute for Nursing Science and Practice, Paracelsus Medical University, Salzburg, Austria (M.T.-G., J.O.).; Department for Clinical Neurosciences and Preventive Medicine, University for Continuing Education Krems, Lower Austria, Austria (Y.T.).; European University of Applied Sciences (EUFH) Campus Rostock, University of Applied Sciences, Rostock, Mecklenburg-West Pomerania, Germany (S. Schulz).; Division for Rehabilitation and Recovery, VASCage Center on Clinical Stroke Research, Innsbruck, Tyrol, Austria (S. Sollereder).

**Keywords:** administration, oral, clinical relevance, deglutition, deglutition disorders, speech-language pathology, stroke

## Abstract

**BACKGROUND:**

International guidelines recommend standardized dysphagia screening in acute stroke, as up to 75% of patients develop poststroke dysphagia. While medication swallowing is also advised for assessment, no validated screening tools or instrumental evidence exist on solid dosage form (SDF) management. Whole tablets are often crushed to reduce aspiration risk, yet the actual risk of aspiration for both forms remains unknown. Despite its widespread clinical use, crushed medication swallowing has never been evaluated using Fiberoptic Endoscopic Evaluation of Swallowing. Closing this evidence gap is essential for guiding safe medication administration in patients with dysphagic stroke.

**METHODS:**

A prospective, single-center, cross-sectional study with an experimental design was conducted on 60 patients with acute stroke (<7 days). The swallowing safety and efficiency of 1 crushed placebo tablet and 3 different types of whole placebo tablets, each administered with an accompanying applesauce bolus, were compared using Fiberoptic Endoscopic Evaluation of Swallowing. The primary outcome was the incidence of unsafe swallowing, as determined by the Penetration-Aspiration Scale. Secondary outcomes included the presence and location of pharyngeal residue associated with each SDF condition.

**RESULTS:**

Of 60 patients with stroke (mean age, 73.4±10.7 years; 55% men) with a median Gugging Swallowing Screen score of 14 indicating moderate dysphagia, 58 patients completed the crushed SDFs trial. Whole SDFs were not swallowed (chewed, spit out, and stopped) in 20 of the 174 trials. All fully swallowed tablets (n=154) were safe, with no penetration or aspiration observed. In contrast, 22 of 154 accompanying boli (14.3%) and 7 of 58 crushed SDFs (12.1%) were rated unsafe, without a significant difference. Crushed SDFs caused significantly more vallecular residues (*P*<0.001).

**CONCLUSIONS:**

These findings highlight the need to reconsider the routine practice of crushing SDFs for patients with dysphagic stroke. A paradigm shift towards using whole SDFs, guided by Fiberoptic Endoscopic Evaluation of Swallowing, could enable more frequent administration, reduce errors, and enhance medication efficacy.

**REGISTRATION:**

URL: https://www.clinicaltrials.gov; Unique identifier: NCT05173051.

Stroke is a leading cause of mortality and disability worldwide.^1^ Poststroke dysphagia (PSD) affects up to 75% of patients, increases the risk of respiratory and nutritional complications, and underscores the need for standardized swallowing assessments.^2,3^ Solid dosage forms (SDFs) pose challenges in PSD, yet robust evidence for modifying them is lacking. Although Fiberoptic Endoscopic Evaluation of Swallowing (FEES) and VFSS (Videofluoroscopic Swallowing Study) are gold standards in swallowing diagnostics, only 4 studies have examined SDF swallowing via FEES, 2 of which included patients with stroke.^4–8^

One of the earliest studies on whole medication intake using FEES was conducted by Carnaby-Mann and Crary,^5^ who assessed swallowing safety, clearance, and residue in 36 patients with subacute dysphagia, including 15 patients with stroke. They found that swallowing conventional tablets without an accompanying bolus required significantly more swallows and prolonged swallowing duration compared with newly designed disintegrating tablets.^5^ A decade later, Schiele et al^4^ evaluated 4 different SDFs in 52 patients with subacute stroke and found an increased risk of penetration and aspiration across all accompanying boli, with texture-modified fluids posing a lower risk than thinner consistencies. Notably, no aspiration of SDFs was observed, and penetration events were not reported.^4^

Despite the clinical relevance of SDF administration in patients with dysphagia, research remains limited, forcing clinicians to rely on experiential knowledge rather than evidence-based guidelines.^9,10^ As a consequence, SDF modifications, such as crushing tablets or opening capsules, are frequently implemented without robust empirical evidence, potentially leading to dose inaccuracies, altered pharmacokinetics, and an increased risk of medication-related complications.^8,9,11–13^

A scoping review identified that 77% of caregivers routinely crush tablets for patients with dysphagia. Similarly, an interview study found that 88% of hospital nurses modify SDFs before administration, with 35% reporting inadequate training.^10,11^ A multicenter study showed increased medication errors and adverse outcomes in this population.^13^ Another survey found that nearly 10% of professionals administer oral medication despite intake restrictions, while a survey of 143 stroke nurses reported universal avoidance of whole tablets in acute PSD.^14,15^

Given the limited evidence on the safety and efficacy of swallowing whole versus modified SDFs in PSD, further research using validated instrumental assessments is warranted. This study aims to systematically evaluate the swallowing safety and efficiency of solid versus crushed SDFs in patients with poststroke dysphagia, providing clinical guidance based on objective, gold-standard methodologies.

## Methods

### Data Availability Statement

The data that support the findings of this study are available from the corresponding author upon reasonable request.

### Study Design and Patient Selection

A prospective, single-center, noninterventional cross-sectional study with an experimental design (within-subject design) was performed from February 2022 to December 2023.

Sixty patients with acute stroke were consecutively enrolled within the first week of stroke onset. The primary outcome was swallowing safety, assessed using the Penetration-Aspiration Scale (PAS), with a PAS score of >2 indicating impaired swallowing safety. Secondary outcomes included swallowing efficiency (Yale Pharyngeal Residue Severity Rating Scale, need for additional boli, and SDFs remaining in the pharynx) and comprehensive swallowing performance (FEES-based classification of dysphagia for solid dosage forms [Fi-prooF]/FEES-based classification of dysphagia for solid dosage forms [crushed; Fi-prooF-c] scale).

The study’s inclusion criteria encompassed patients (1) <7 days after acute stroke, (2) aged between 40 and 100 years, (3) with a Gugging Swallowing Screen score of <20 points (indicating dysphagia), (4) a need for an instrumental assessment, and (5) a signed informed consent. To reflect clinical reality, patients with nasogastric tubes were included, as evidence on their potential impact on swallowing safety and efficiency remains limited.

Patients meeting any of the following conditions were excluded from the study: (1) those with neurodegenerative diseases, (2) within 2 hours post-extubation, (3) with a tracheostomy, (4) with chronic obstructive pulmonary disease, (5) preexisting dysphagia, (6) pill dysphagia in medical history, and (7) all patients with contraindications for conducting an FEES evaluation.

Before the FEES examination, patients and dedicated nurses were independently asked whether they considered swallowing whole tablets feasible and safe.

**Figure 1.**
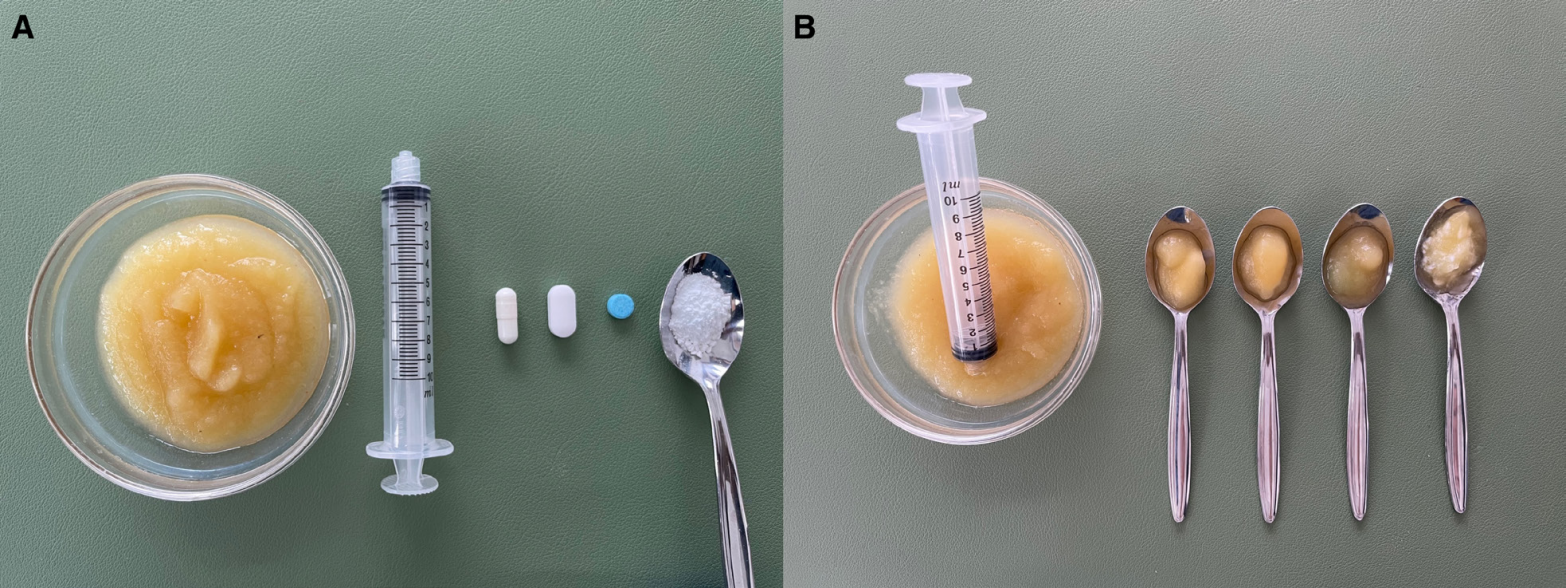
**Presentation of the used placebos. A**, Whole and crushed. **B**, Mixed with 3 mL of applesauce.

A structured FEES evaluation was performed using whole and crushed placebo tablets within the first week of stroke onset. The placebo tablets were selected based on the 51 most prescribed solid medications in the stroke unit over 1 year. As a result, 1 small, round blue tablet (diameter: 8 mm; manufacturer: Winthrop Arzneimittel GmbH, Zentiva Pharma GmbH, Germany; PZN: 03935636, P-Tabletten blau Lichtenstein), an oblong tablet (length: 17 mm and width: 8.2 mm, manufacturer: Fagron GmbH & Co KG; PZN: 00921088), and a size-2 filled capsule (custom-made, in-house pharmacy) were chosen (Figure 1).

For the swallowing examination requiring tablet crushing, an oblong 17 mm×8.2 mm placebo (Fagron) was used to simulate clinical practice. A turning mortar ensured standardized crushing. The sequence in which the 3 whole placebo tablets were administered was randomized using dedicated software.^16^ Applesauce was selected as an accompanying bolus, consistent with clinical practice.^15^ The in-house applesauce was subjected to a consistency test according to the measurement methods of the International Dysphagia Diet Standardization Initiative and classified as level 4.^17^

### Setting

The research was conducted in a specialized stroke unit with 6 dedicated beds for patients with stroke. Comprehensive clinical data were collected as part of the clinical routine, including the National Institutes of Health Stroke Scale, the modified Rankin Scale, the Gugging Swallowing Screen, the Dysphagia Severity Rating Scale, the Oral Health Assessment Tool, and the patients’ stroke diagnoses and age.^18–22^ All relevant data, including findings from FEES, were systematically collected, meticulously organized, and securely stored in accordance with research standards and data protection guidelines.

### FEES-Procedure and Measurement

The study equipment comprised a 4-mm diameter flexible fiberoptic rhino laryngoscope (model/type: RS1-PAL; RS1-NTSC; RX1; manufacturer: orlvision GmbH), a light source (rpLight-LED; Rehder/Partner GmbH, Hamburg, Germany), and the rpSzene Panel-PC and rpSzene software (rpSzene; Rehder/Partner GmbH).

The FEES investigation was performed bedside or in a wheelchair. The laryngoscopic examination was performed by a team of 3 speech and language therapists with several years of experience in this field. First, the patients were given 3 initial teaspoons of applesauce (baseline investigation), followed by a crushed 17-mm placebo mixed with 3 mL of applesauce. The study was discontinued if a PAS score of ≥5 was recorded in any baseline or crushed placebo trials, indicating a high risk of aspiration.

**Figure 2.**
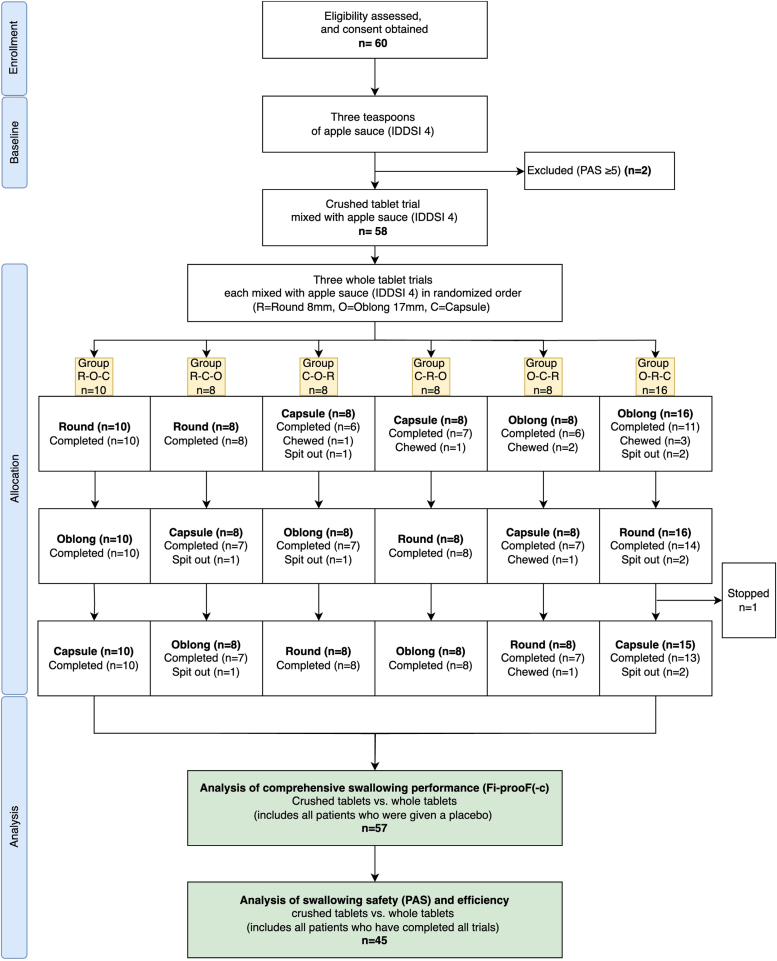
**Flowchart of patient enrollment and allocation to the randomized Fiberoptic Endoscopic Evaluation of Swallowing trial groups.** Fi-prooF-c indicates Fiberoptic Endoscopic Evaluation of Swallowing–based classification of dysphagia for solid dosage forms (crushed); IDDSI, International Dysphagia Diet Standardization Initiative; and PAS, Penetration-Aspiration Scale.

The 3 different placebos were administered with 3 mL of applesauce on a teaspoon in the random sequence specified for each patient. Patients who could not swallow the placebo on the first attempt were asked if they needed another bolus of applesauce. The study was halted for safety reasons if a whole placebo tablet received a PAS score of ≥3 or if there were any other concerns about continuing. The patient enrollment process and the randomized sequence of medication administration are described in detail in Figure 2.

### Measurement of Swallowing Safety (PAS)

The 8-point PAS was used to evaluate swallowing safety across all trials, including baseline, crushed placebo, and whole placebo boli, with separate assessments for tablets and the accompanying boli in the whole placebo trials.^23^ For data analysis, the scale was dichotomized into PAS score of 1 to 2 and PAS score of 3 to 8, indicating a low versus moderate to high risk of penetration aspiration, respectively.

### Measurement of Swallowing Efficiency

The Yale Pharyngeal Residue Severity Rating Scale was used to categorize the quantity of residues present in the valleculae or piriform sinuses of all applesauce boli, providing an objective measure to assess swallowing efficiency.^24^ Whole placebo tablets that remained in the valleculae, piriform sinuses, or pharynx were independently documented, as were crushed placebo tablets lingering on the base of the tongue.

Furthermore, the count of additional boli required for the whole placebos was recorded per tablet. Spit-out and chewed placebos were recorded specifically, and the trials were excluded from the final safety and efficiency analysis (Figure 2).

### Measurement of Comprehensive Swallowing Performance

The FEES-based classification of dysphagia for SDFs (Fi-prooF) scale was introduced for this study to integrate swallowing safety and efficiency into a single comprehensive scale. It builds upon a combined swallowing safety and efficiency scale developed by Buhmann et al^6^ and on additional refinements introduced by Labeit et al^7^ in patients with Parkinson disease. The resulting scale consists of 4 subtests, SDF efficiency, SDF safety, accompanying bolus efficiency, and accompanying bolus safety, each assessed on its own ordinal scale with severity levels ranging from 1 (no impairment) to 4 (severe impairment). The categories include established measures such as the PAS score and the Yale Pharyngeal Residue Severity Rating Scale while also considering spillage, chewing, dissolution, and spitting out of SDFs. The worst score observed in these 4 categories determines the overall severity score, ranging from 1 to 4 (1=no impairment, 2=mild impairment, 3=moderate impairment, and 4=severe impairment). To date, the scale has not been validated in patients with stroke.

For assessment of the swallowing performance of crushed SDFs, the Fi-prooF scale was adapted by the principal investigator. The newly developed 4-point ordinal scale differentiates according to the same safety and efficiency categories and is referred to as Fi-proof-c (Tables S1 and S2).

The evaluation of FEES results relevant to the study was performed by the principal investigator using the recorded video, employing the slow-motion and frame-by-frame capabilities of the video analysis software.

### Interrater Reliability of PAS and Fi-prooF Scales

After study completion, 44 swallowing attempts were randomly selected for blinded interrater reliability assessment by an independent rater with over 10 years of FEES experience. Weighted Cohen κ (quadratic) for the overall Fi-prooF scale was 0.53, indicating moderate agreement between raters.

For the dichotomized PAS values of the SDF swallowing trials, Cohen κ could not be calculated due to a lack of variance, as nearly all PAS scores were constant across both raters (predominantly scored as 1; percent agreement=97.6%). For the PAS scores of the accompanying boli, Cohen κ revealed moderate agreement between the raters (κ=0.50; percent agreement=88.6%) based on the same interpretative framework.

### Study Size

No formal sample size calculation was conducted due to the lack of data on the prevalence of swallowing difficulties with whole and crushed SDFs in patients with acute PSD, as well as the absence of evidence regarding their impact on aspiration or penetration risk.

### Statistical Methods

Descriptive statistics, along with diagrams and tables, were generated using both Excel (Excel for Mac, version 16.89.1) and SPSS (IBM SPSS Statistics, version 29.0.2.0).

The Cochran Q test was initially planned to analyze differences in swallowing safety outcomes using binary PAS categorization (PAS score of 1–2 versus PAS score of 3–8) across crushed and whole placebo trials. However, the test could not be applied due to a lack of variability in the PAS data (see results). The Cochran Q test was solely applied to both accompanying boli (placebo mixed with 3 mL of applesauce) and baseline boli (3 teaspoons of applesauce), where sufficient variability was present.

To analyze differences in pharyngeal residues between the 2 pharyngeal regions (valleculae and piriform sinuses) during applesauce swallows, consisting of 3 baseline swallows, 3 accompanied boli, and 1 crushed bolus, the Wilcoxon signed-rank test was applied, resulting in 7 pairwise comparisons. Bonferroni correction was applied (α_adj_=0.00714) to adjust for multiple comparisons.

The McNemar test was used to evaluate paired differences in the retention of whole SDFs between the valleculae and the piriform sinuses. In a subgroup analysis of vallecular residues, crushed medications were compared with SDFs. For this, the Yale Pharyngeal Residue Severity Rating Scale, originally a 5-point ordinal scale, was dichotomized: Scores of 1 to 2, indicating no or minimal residue, were classified as low residue, while scores of 3 to 5, indicating mild to severe residue, were classified as high residue, reflecting the presence of a small to large tablet in the valleculae. The Cochran Q test was used to assess differences in residue presence between the crushed medications and whole tablets.

To analyze differences in the comprehensive swallowing performance, as measured by the Fi-prooF-c scales, between the 4 placebo trials (8 mm, capsule, 17 mm, and crushed) in participants who received all placebo tablets (n=57), the Friedman test with post hoc rank-based pairwise comparisons was applied. To account for the increased risk of type I errors associated with multiple comparisons, post hoc analyses were adjusted using the Bonferroni correction.

### Ethical Approval and Data Protection

The study was approved by the Province of Lower Austria Ethics Committee (approval number: GS4-EK-4/698-2021). Written informed consent was obtained from all participants before inclusion in the study. Data were anonymized and securely stored in accordance with institutional and national data protection guidelines. The data that support the findings of this study are available from the corresponding author upon reasonable request.

This study adheres to the STROBE guidelines for reporting observational studies (Table S3).

## Results

### Study Population

Sixty patients with acute stroke with dysphagia (mean age, 73.4±10.7 years; 33 men) were included (Table 1). Nurse-patient agreement on pill swallowing ability was 53.3%.

**Table 1.**
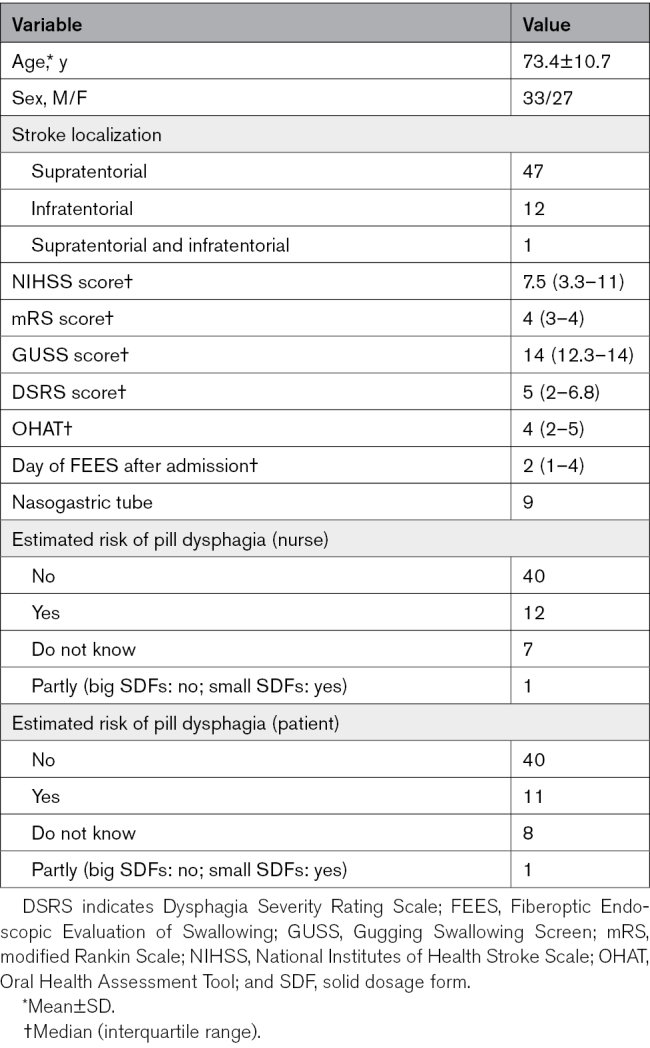
Demographic Data of 60 Patients With Stroke

### Outcome Data

#### Primary Outcome: Swallowing Safety

Swallowing safety rates, categorized as PAS score of 1 to 2 (safe) versus PAS score of 3 to 8 (unsafe), are summarized for all tablet trials in Table 2. Swallowing safety was consistently high across all trials, with the number of trials with PAS scores of 1 to 2 ranging from 41 to 55 cases. Notably, all SDFs were classified as safe, precluding the use of statistical tests. Accompanying boli were considered unsafe (PAS score of 3 to 8) in 6 cases for the 8-mm tablets, 7 cases for the 17-mm oblong tablets, and 9 cases for the capsules.

**Table 2.**
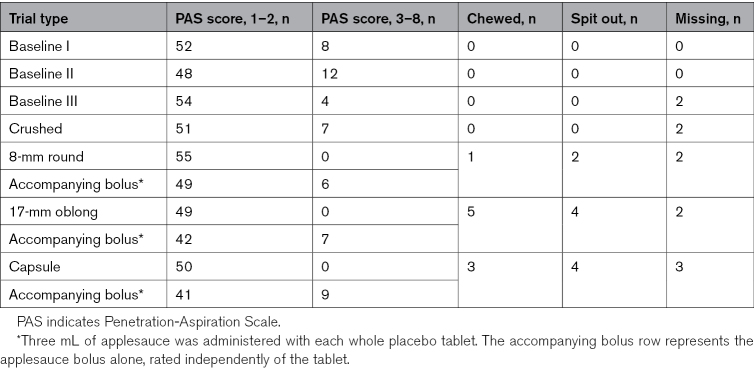
PAS Scores of Baseline, Crushed, and Whole Placebo Trials (Low [PAS Score, 1–2] Versus Medium to High [PAS Score, 3–8] Penetration/Aspiration Risk; N=60)

When comparing the dichotomized PAS scores of the 3 baseline swallows with the 3 accompanied boli of the 8 mm, 17 mm, and capsule forms, no significant differences were detected (Cochran Q=3.738; df=5; *P*=0.588). Similarly, no significant differences were identified between the PAS scores of crushed SDFs and the accompanied boli (Cochran Q=2.442; df=3; *P*=0.486).

#### Swallowing Efficiency: Residues

Residue levels in the valleculae and piriform sinuses were compared for each bolus type (baseline, crushed, and accompanied boli) across 45 patients who completed all test swallows. The results revealed significantly higher residue levels in the valleculae compared with the piriform sinuses for all 7 swallows (3 baseline swallows, 3 accompanied boli, and 1 crushed bolus; all *P*<0.001). To account for multiple comparisons, a Bonferroni correction was applied, adjusting the significance threshold to α_adj_=0.006.

Whole SDFs were more frequently retained in the vallecular space than in the piriform sinuses, as analyzed using the McNemar test. This difference was significant for the 17 mm tablet (*P*=0.021) but not for the capsule (*P*=0.125) or the 8-mm tablet (*P*=0.219). The number of patients requiring additional boli to facilitate medication transit and pharyngeal clearance varied across bolus types, ranging from 4 (8-mm placebo) to 8 (placebo capsule) and 15 (17-mm placebo), with most patients requiring only 1 additional bolus.

### Vallecular Residue Subgroup Analysis

Based on the finding that residues were consistently higher in the vallecular than in the piriform region, a subgroup analysis was conducted focusing on vallecular residues.

Vallecular residue severity differed significantly across bolus types (Friedman, χ² [6]=40.183; *P*<0.001). Post hoc pairwise comparisons with Bonferroni correction demonstrated that crushed SDFs caused significantly higher residue levels in the valleculae compared with those of the accompanying boli administered during whole SDF trials (8-mm *P*_adj_=0.001; 17-mm *P*_adj_=0.004; and capsule *P*_adj_=0.048).

To compare vallecular pharyngeal residues between SDF and crushed SDFs, the Yale Pharyngeal Residue Severity Rating Scale was dichotomized to enable a direct comparison of residue presence (score of 3–5) or absence (score of 1–2) and align it with the binary classification of tablets remaining in the vallecular space. The Cochran Q test revealed a highly significant overall difference in pharyngeal residue severity among the tested placebo types (Q=174.889; df=10; *P*<0.001), indicating substantial variation across the groups. Pairwise comparisons with Bonferroni correction further demonstrated that crushed medications resulted in significantly higher vallecular residue severity compared with all types of whole SDFs remaining in the valleculae (all *P*_adj_<0.001).

**Figure 3.**
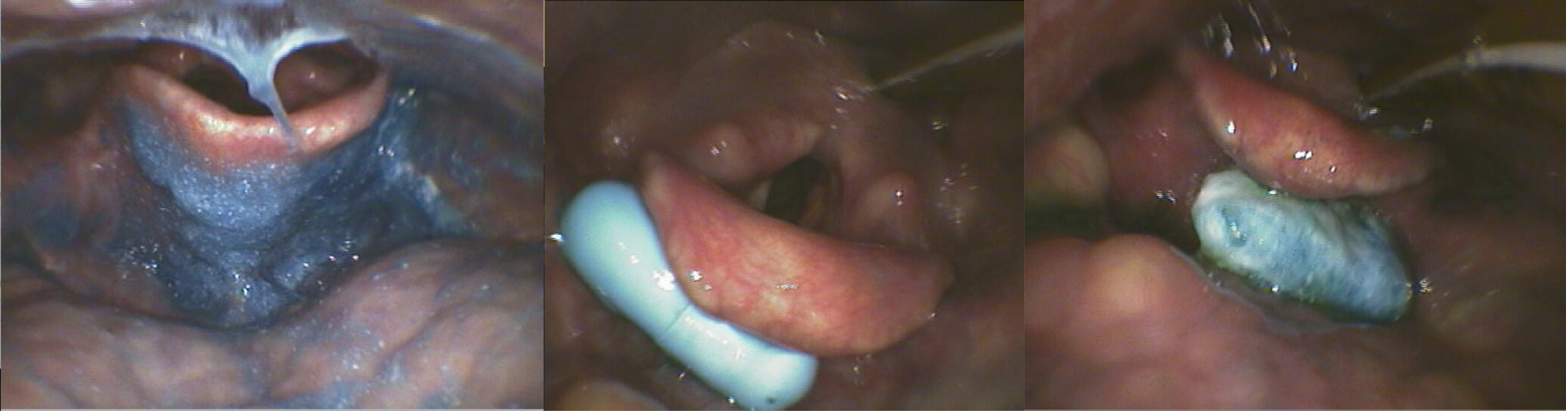
Crushed vs whole solid dosage forms remaining in the vallecular space.

In addition, residues of crushed SDFS were also found in other regions: 27 patients exhibited residue at the base of the tongue, 16 patients had residue at the posterior pharyngeal wall, and 11 patients presented with residue in both regions simultaneously (Figure 3).

#### Comprehensive Swallowing Performance (Safety and Efficiency)

The Fi-prooF-c severity scales revealed notable differences in impairment levels across the tested bolus types (Table 3). The difference between the 4 groups was statistically significant (Friedman test χ² [3]=15.632; *P*=0.001). Post hoc pairwise comparisons showed significant differences between the 8-mm placebo and the crushed placebo (*P*_adj_=0.022). In addition, a significant difference was observed between the 8- and 17-mm trials (*P*=0.011); after adjustment for multiple comparisons with Bonferroni, this difference was no longer significant (*P*_adj_=0.067).

**Table 3.**
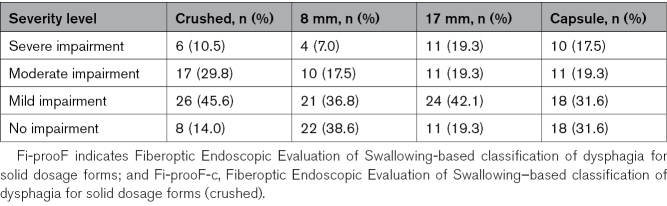
Patient Distribution Across Severity Categories of the Overall Fi-prooF(-c)Scales Across All Placebo Trials (Crushed, 8 mm, 17 mm, Capsule; n = 57)

## Discussion

The findings of this study challenge the widespread clinical practice of routinely crushing medications in acute PSD by presenting objective evidence on the safety and efficiency of swallowing whole SDFs. The National Institutes of Health Stroke Scale score range (1–21; median, 7.5) reflects a predominantly moderately impaired stroke cohort, consistent with clinical practice where patients with high scores are often not referred for swallowing evaluation in the acute phase^25^; in addition, severe dysphagia cases (puree bolus PAS scores ≥5) were excluded based on FEES for patient safety. These findings provide valuable evidence applicable to patients with moderate neurological impairments and emphasize the importance of FEES as a reliable tool in guiding clinical decision-making. While previous studies have primarily examined the swallowing safety of whole SDFs in subacute stroke populations,^4,5^ this study is the first to systematically compare whole versus crushed SDFs in acute PSD using instrumental assessment. This distinction is critical, as early medication administration is a pivotal factor in stroke management.

A key outcome of this study was the confirmation that whole SDFs themselves pose no safety risk to patients with PSD, with 94.8% of trials rated as safe (PAS score, 1–2) and a complete absence of any penetration or aspiration (PAS score, 3–8) observed. This aligns with prior literature, showing no evidence of aspiration with whole SDFs in subacute stroke.^4,5,7^ The high rater agreement in the evaluation of PAS scores supports the robustness of these findings. Although accompanying boli occasionally led to a PAS score of 3 to 8 events, these were not significantly different from baseline swallows and remained within a low-risk range (≤18% unsafe). Applesauce (International Dysphagia Diet Standardization Initiative 4), widely used in clinical practice, proved to be a safe and suitable accompanying bolus for whole SDFs in this study, consistent with previous research supporting pureed over liquid consistencies for safer swallowing.^4–7^

In some cases, larger tablets required additional boli, suggesting that adjusting bolus volume could optimize swallowing performance.

Crushed medications significantly increased in pharyngeal residue, mainly in the valleculae, compared with whole tablets or accompanying boli. In contrast, accompanying boli did not lead to a significant increase in residue compared with baseline assessments, a finding that differs from the results of Schiele et al,^4^ where liquids such as milk were associated with increased residue in the valleculae and pyriform sinuses. When safety and efficiency were combined, patients showed a significantly better swallowing performance of 8-mm tablets compared with crushed medications. These findings highlight the clinical challenges of crushed medications, which impair swallowing efficiency, increase residue-related complications, and may delay dissolution, affecting bioavailability.^11,26^

The absence of aspiration events in this study contrasts with findings from research using thinner consistencies, reinforcing the importance of appropriate bolus texture selection.^4,5^ In addition, the discrepancy in residue patterns demonstrates the need for further investigation into how different consistencies interact with various medication forms.

The results of this study support a paradigm shift in PSD medication management, advocating for an individualized, FEES-guided approach to prevent unnecessary tablet modifications.

### Limitations

While this study provides robust evidence supporting the safety of whole SDFs, certain limitations must be acknowledged. The single-center design may limit generalizability, and the exclusion of patients with severe comorbidities or prior dysphagia reduces applicability to more complex cases. In addition, selection bias may be present, as only patients capable of providing consent were included.

A potential performance bias exists, as the primary investigator conducted all FEES examinations. To mitigate this, 44 videos were independently rated, with interrater reliability assessed. The moderate agreement observed with the Fi-ProoF-c may reflect the complexity of the scales and the need for specific training. Further validation and standardization are required to improve consistency across clinical settings.

### Conclusions

This study provides the first instrumental evidence that crushing SDFs does not increase swallowing safety in patients with PSD. Findings confirm that whole SDFs, when administered with applesauce (International Dysphagia Diet Standardization Initiative 4) and assessed via FEES, are safe in acute PSD, with no increased aspiration risk. In contrast, crushed medications result in significantly higher pharyngeal residue, particularly in the valleculae, which may delay drug absorption and increase the risk of local irritation, bacterial colonization, or aspiration of dissolved medication.

These results challenge the routine modification of SDFs and highlight the need for evidence-based guidelines in PSD medication management. Future research should not only validate these findings in larger, multicenter cohorts but also explore alternative drug formulations, such as orodispersible tablets, which may enhance swallowing safety and improve the feasibility of administration in patients with PSD.

## Article Information

### Acknowledgments

The authors want to appreciate the contribution of NÖ Landesgesundheitsagentur, a legal entity of the University Hospitals in Lower Austria and Karl Landsteiner University of Health Sciences, Krems, Austria, for providing the institutional and organizational framework that enables this research. The authors acknowledge the use of automated writing assistance tools (ChatGPT and Grammarly) for language refinement. The authors take full responsibility for the accuracy and originality of this article.

### Sources of Funding

Open access publication was supported by the Open Access Publishing Fund of Karl Landsteiner University of Health Sciences, Krems, Austria. No additional financial support was received from funding agencies, commercial organizations, or nonprofit institutions.

### Disclosures

None.

### Supplemental Material

Tables S1–S3
